# Evidence of intensification of pyrethroid resistance in the major malaria vectors in Kinshasa, Democratic Republic of Congo

**DOI:** 10.1038/s41598-023-41952-2

**Published:** 2023-09-07

**Authors:** Daniel Nguiffo-Nguete, Leon M. J. Mugenzi, Emile Zola Manzambi, Magellan Tchouakui, Murielle Wondji, Theofelix Tekoh, Francis Watsenga, Fiacre Agossa, Charles S. Wondji

**Affiliations:** 1 Centre for Research in Infectious Diseases (CRID), P.O. Box 135091, Yaoundé, Cameroon; 2 Institut National de Recherche Biomédicale, Kinshasa, Democratic Republic of Congo; 3 Department of Vector Biology, Liverpool School of Tropical Medicine, Pembroke PlaceLiverpool, L35QA UK; 4 Faculty of Sciences, University of Buea, Buea, Cameroon; 5 International Institute of Tropical Agriculture (IITA), P.O. Box 2008, Yaoundé, Cameroon

**Keywords:** Infectious diseases, Public health

## Abstract

Assessing patterns and evolution of insecticide resistance in malaria vectors is a prerequisite to design suitable control strategies. Here, we characterised resistance profile in *Anopheles gambiae* and *Anopheles funestus* in Kinshasa and assess the level of aggravation by comparing to previous 2015 estimates. Both *species* collected in July 2021 were highly resistant to pyrethroids at 1×, 5× and 10× concentrations (mortality < 90%) and remain fully susceptible to bendiocarb and pirimiphos methyl. Compared to 2015, Partial recovery of susceptibility was observed in *A. gambiae* after PBO synergist assays for both permethrin and α-cypermethrin and total recovery of susceptibility was observed for deltamethrin in 2021. In addition, the efficacy of most bednets decreased significantly in 2021. Genotyping of resistance markers revealed a near fixation of the L1014-*Kdr* mutation (98.3%) in *A. gambiae* in 2021. The frequency of the 119F-GSTe2 resistant significantly increased between 2015 and 2021 (19.6% vs 33.3%; *P* = 0.02) in *A. funestus*. Transcriptomic analysis also revealed a significant increased expression (*P* < 0.001) of key cytochrome P450s in *A. funestus* notably *CYP6P9a*. The escalation of pyrethroid resistance observed in *Anopheles* populations from Kinshasa coupled with increased frequency/expression level of resistance genes highlights an urgent need to implement tools to improve malaria vector control.

## Introduction

Malaria remains a major public health concern in low-income countries. Democratic Republic of Congo (DRC) has the second highest burden in the world with approximately 12% of malaria cases and 13.2% of all death^1,2^. Malaria prevention relies extensively on insecticide-based intervention such as long-lasting insecticide-treated mosquitoes nets (LLINs). Unfortunately, the development of insecticide resistance is reducing the efficacy of these control methods^3–5^. Two main resistance mechanisms have been described in malaria vectors, namely the target site insensitivity^6–8^ and metabolic resistance. Previous studies conducted in DRC have reported an increasingly widespread resistance to pyrethroids, carbamates and organochlorines mainly in the major malaria vectors *Anopheles gambiae*^4,9^ and *Anopheles funestus*^5^. High resistance intensity is gradually reported on the continent in both species such as in Uganda^10^, Malawi^2^, Ghana^11^ with risk of further jeopardising the current control tools. Temporal assessment of change in this resistance level between years is a good approach to capture the extent of the resistance escalation as done in Malawi^2^ beside resistance intensity generated by comparing exposure to 1×, 5× and 10×. In DRC, since a thorough assessment was performed in 2015 in Kinshasa in both *A. funestus* and *A. gambiae* populations showing the resistance, this offers an excellent choice to measure the extent of resistance aggravation in this part of the country. Therefore, this study was conducted in 2021 to capture the increase resistance intensity six years after that initial assessment done in 2015 and published by^5^ in the same area in both *A. funestus* and *A. gambiae* and to use the WHO method to measure resistance intensity and assess resistance escalation molecularly.

## Results

### Species composition

A total of 297 adult female *Anopheles* mosquitoes (55.5% *A. gambiae* s.l and 44.5% *A. funestus* s.s) were collected indoor in Ndjili-Brasserie. All samples (60) from the *A. funestus* group were molecularly confirmed as being *A. funestus* s.s. while 59 out of the 60 F_0_ (98.33%) *A. gambiae* s.l, belonged to *A. gambiae* s.s. species and one (1.67%) was *A. coluzzii*.

### *Plasmodium* infection rate

A total of 101 *A. funestus* was tested for *Plasmodium* infection using TaqMan. The analysis revealed 8.9% (9/101) of mosquitoes infected with *Plasmodium falciparum* only with 7 out of these 9 confirmed by nested PCR as *P. falciparum (Pf* +*).* Infection rate was higher in *A. gambiae* s.l. with 25.2% (29/115) of mosquitoes infected with *P. falciparum,* 0.8% (1/115) infected with *Plasmodium ovale*, *Plasmodium vivax*, and/or *P. malariae* (OVM+) and 4% (5/115) were co-infected (Pf+/OVM+). The nested PCR failed to confirm 5.2% (2/38) Pf + infections and 40% (2/5) mix infections (Pf+/OVM+). Overall, the nested PCR confirmed 36 mosquitoes infected with *Pf*+, one (1) infected with *P. malariae*, one (1) infected with *P. falciparum* + *P. ovale* and *2* infected with *P. falciparum* + *P. malariae.*

Compared to the *Plasmodium* infection rate results obtained in 2015, we broadly observed a reduction in the *Plasmodium* infection rate in the *A. funestus* population (30% in 2015 vs. 8.9% in 2021; *P* = 0.0003), and not difference in *A. gambiae* population (41.2% in 2015 vs. 30.4% in 2021; *P* = 0.09).

### Insecticide susceptibility assays

#### Bioassays with the discriminating concentration 1× (DC) in *A. gambiae* s.l. and *A. funestus* s.s

**Figure 1:**
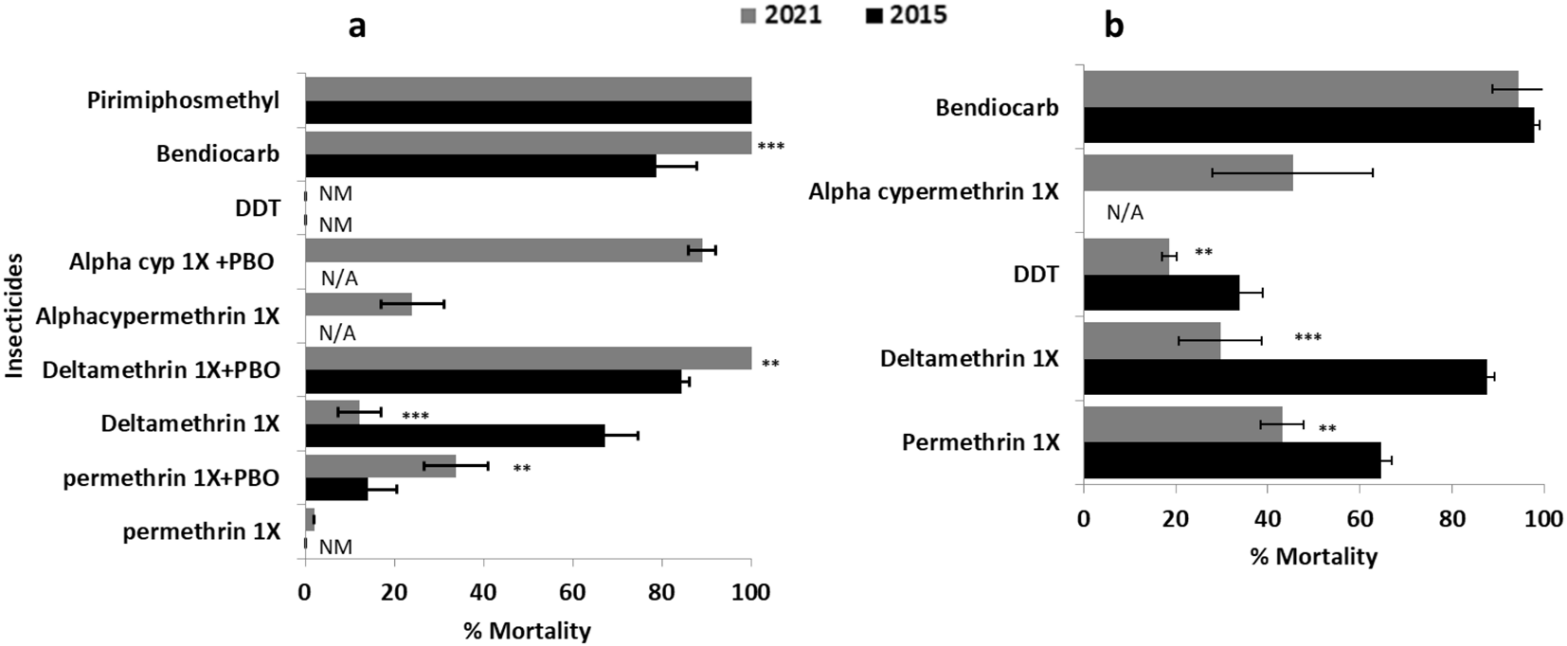
Susceptibility profile of *Anopheles gambiae* (**a**) and *Anopheles funestus* (**b**) population in Kinshasa in 2015 and 2021 using World Health Organization insecticide susceptibility tube assays. NM, no mortality.

The F_1_ progeny of *A. gambiae* s.l. from this field population showed an extremely high resistance to type I and type II pyrethroids. For permethrin (Type I), mortality was 2.1 ± 1.2%. For deltamethrin and α-cypermethrin (Type II), mortality was 12.2 ± 4.9% and 23.6 ± 7.3% respectively. High resistance was also observed for the organochlorine (DDT) with 0% mortality. However, full susceptibility was observed with the organophosphate (pirimiphos-methyl) and carbamate (bendiocarb) with a 100% mortality rate (Fig. 1a).

The *A. funestus* s.s. population was resistant to permethrin (43.1% ± 4.7%), deltamethrin (28.7% ± 5.9%), α-cypermethrin (45.5% ± 17.5%) and DDT (18.6% ± 1.6%), but only moderately resistant to bendiocarb (94.4% ± 1.2%) (Fig. 1b). There were not enough of mosquitoes to perform pirimiphos-methyl bioassay test with *A. funestus*.

#### Bioassays with pyrethroid 5× and 10× DC in *A. funestus* s.s. and *A. gambiae* s.l

**Figure 2:**
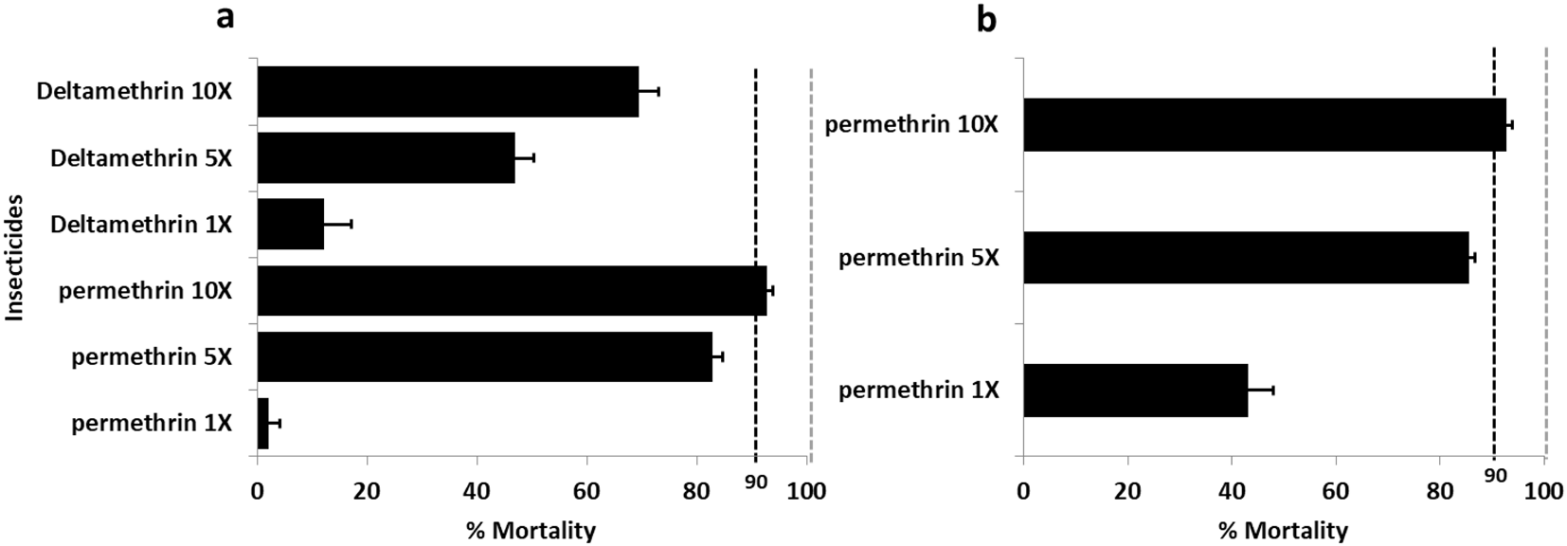
Susceptibility profile of *Anopheles gambiae* (**a**) and *Anopheles funestus* (**b**) population in Kinshasa using resistance intensity with 1×, 5× and 10× the diagnostic concentrations of permethrin (0.75%) and deltamethrin (0.05%) World Health Organization insecticide susceptibility tube assays.

Bioassays were carried out with 5× DC and 10× DC of permethrin (3.75% and 7.5%) and deltamethrin (0.2% and 0.5%) to assess the resistance intensity. According to WHO criteria this population is highly resistant because we have mortality < 98% at 10×*.* Hence, *A. funestus* s.s exhibited a mortality rate of 87.5% ± 1.9% and 92.6 ± 2.7% to permethrin 5× and 10× respectively (Fig. 2b). *Anopheles gambiae* s.l showed a mortality rate of 82.7% ± 1.9% and 92.7% ± 1.0% respectively with permethrin 5× and 10× (Fig. 2a). However, higher intensity resistance was observed with deltamethrin 5× and 10× with respective prevalence of 46.9% ± 3.4% and 69.4% ± 3.3% (Fig. 2a).

#### PBO synergist assays with *A. gambiae* s.l

Because of the limited number of *A. funestus*, synergist assays were carried out only with *A. gambiae *s.l*.* The synergist assay results showed a slight recovery of susceptibility to deltamethrin and α-cypermethrin. An increased mortality was observed after PBO exposure from 2.0 ± 1.2 to 33.8 ± 7.2% (χ^2^ = 32.0; *P* < 0.0001) for permethrin and from 23.6 ± 7.3 to 88.4 ± 2.5% (χ^2^ = 95.5; *P* < 0.0001) mortality for α-cypermethrin (Fig. 1a). However, PBO led to full recovery of susceptibility to deltamethrin from 12.2 ± 4.9 to 100% mortality (χ^2^ = 77.5; *P* < 0.0001). These results show that cytochrome P450s are playing a greater role in the escalation of resistance to type II pyrethroids (deltamethrin and alphacypermethrin) than to type I (permethrin) in *A. gambiae* population from NDjili.

#### Aggravation of pyrethroid resistance in *A. gambiae* s.l. and *A. funestus* s.s. between 2015 and 2021

Bioassay results showed a significantly overall increase resistance intensity to pyrethroids and carbamates in 2021 compared to 2015. The mortality rate of *A. gambiae* after exposure to deltamethrin reduces from 67.2% in 2015 to 12.1% in 2021 (χ^2^ = 58.1 *P* < 0.0001). (Fig. 1a). In contrast, increase mortality rate was observed for the Bendiocarb (78.7% vs. 100%; χ2 = 16.1 *P* < 0.0001). However, no difference in mortality was observed when we compared mosquitoes exposed to permethrin 1× in 2015 and 2021 (0.0% vs. 2.0%; *P* = 0.2) (Fig. 1a).

In *A. funestus*, a significant reduction in mortality rate was noticed between 2021 and 2015 for DDT (33.8% vs. 18.6%; χ^2^ = 18.7, *P* < 0.001), deltamethrin (64.6% vs. 29.6%; χ^2^ = 18.7, *P* < 0.0001), and Permethrin (64.6% vs. 43.1%; χ^2^ = 6.9, *P* = 0.008) (Fig. 1b) while no statistical difference was observed in *A. funestus* population exposed to bendiocarb (97.1% vs. 94.4%; χ^2^ = 0.6, *P* = 0.4).

#### Bioefficacy of LLINs using cone assays in *A. funestus s.s.* and *A. gambiae *s.l

**Figure 3:**
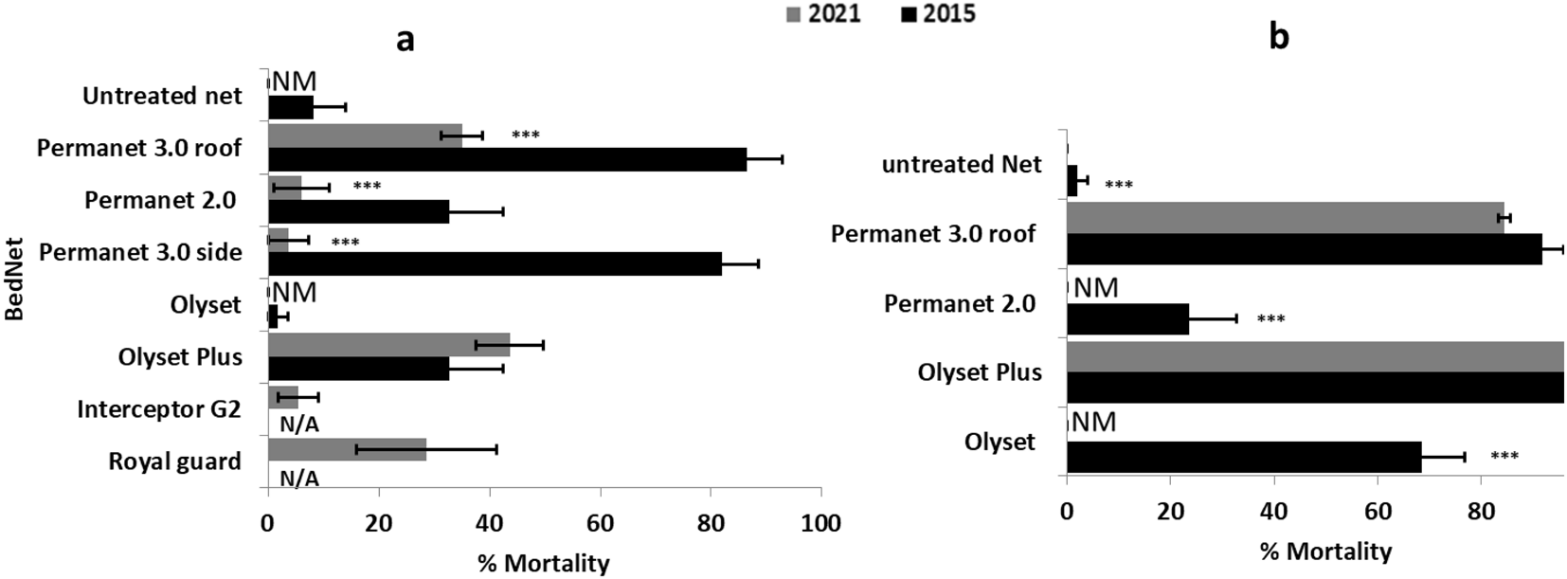
Bioefficacy of *Anopheles gambiae* (**a**) and *Anopheles funestus* (**b**) from Kinshasa in 2015 and 2021 using different long-lasting insecticidal nets; NM, No Mortality; N/A, not applicable.

Low efficacy was recorded against most of the nets tested in *A. funestus* except with Olyset plus and PermaNet 3.0 roof. The mortality rate was 0% for Olyset and PermaNet 2.0. However, Olyset Plus, and PermaNet 3.0 roof (PBO-based nets) did not show a difference of efficacy between 2015 and 2021 (Fig. 3b).

In *A. gambiae* s.l., a lower efficacy was observed for all the nets tested with mortality rate of 0.0% ± 0.0%, 6.1 ± 3.1%, 3.6 ± 3.6% and 5.4 ± 3.6% respectively for Olyset, PermaNet 2.0, PermaNet 3.0 side and Interceptor (α-cypermethrin-based net) (Fig. 3a). The PBO-based nets showed a significant increased efficacy with 43.6 ± 6.0% for Olyset Plus and 26.3 ± 9.1% for PermaNet 3.0 roof. Royal guard (α-cypermethrin + pyriproxyfen), a new generation net, showed mortality rate of 28.7 ± 12.7 (Fig. 3a).

As observed with WHO tube tests, cone assays for bed net efficacy revealed also a significant reduction in mortality rate between 2021 and 2015 in *A. gambiae* mosquitoes after exposure to PermaNet 2.0 (32.7% vs. 6.1%; χ^2^ = 7.4 *P* = 0.007), PermaNet 3.0 side (82% vs. 3.6%; χ^2^ = 35.8, *P* < 0.0001) and PermaNet 3.0 roof (86.5% vs. 26.3%; χ^2^ = 16.2; *P* < 0.0001) and an increase in mortality with Olyset Plus (43.6% vs 32.7%; χ^2^ = 1.2, *P* = 0.25) (Fig. 3a). In *A. funestus*, we recorded the same decrease in mortality with PermaNet 2.0 (24% vs. 0.0%; χ^2^ = 5 *P* = 0.02), and Olyset (68% vs. 0%; χ^2^ = 24.8, *P* < 0.0001) (Fig. 3b). They were no difference between PermaNet 3.0 roof (91.9% vs. 84.5%; χ^2^ = 0.3, *P* = 0.5).

#### Genotyping of insecticide resistance markers in *A. funestus*

**Figure 4:**
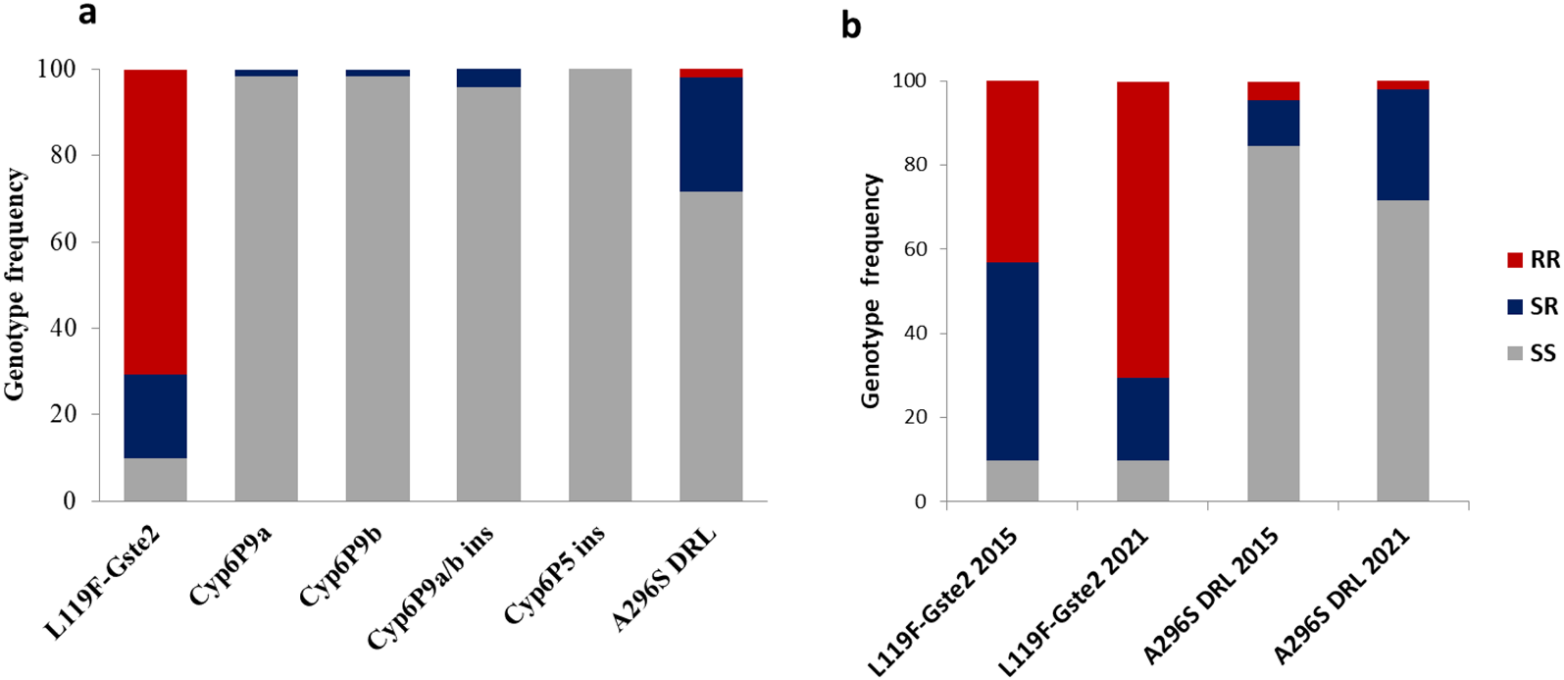
Genotype distribution for key resistance markers (**a**) and genotype comparison between *Anopheles funestus* F0 females collected in 2015 and 2021 (**b**).

In *A. funestus* s.s., the A296S-RDL mutation which confers resistance to dieldrin, was detected. The 296S-resistant allele (R) frequency was low (16.7%) with genotype frequency of 26.4% RS and 71.6% SS and 2% RR (Fig. 4a). This frequency was not different (*P* = 0.13) compared to 2015 (10%). In contrast, the high allelic frequency of the 119F-*GSTe2* significantly increased in 2021 (80.4%) compared to 2015 (66.6%) (*P* = 0.02) with 70.5% of the individuals homozygote resistants’ (RR), 9.8% of homozygous susceptible (SS) and 19.6% heterozygous (RS) (Fig. 4b). The, *Cyp6P9a_R* and *Cyp6P9b_R* resistance markers conferring pyrethroid resistance were for the first time detected although at low frequency: 1.7% and 1.8% respectively in contrast to 2015 where they were completely absent (Fig. 4a). The 6.5 kb insertion conferring the resistance to pyrethroid in the malaria vector remains absent in this population (100% SS).

#### Changes in the frequency of insecticide resistance markers in *A. gambiae* between 2021 and 2015

**Figure 5:**
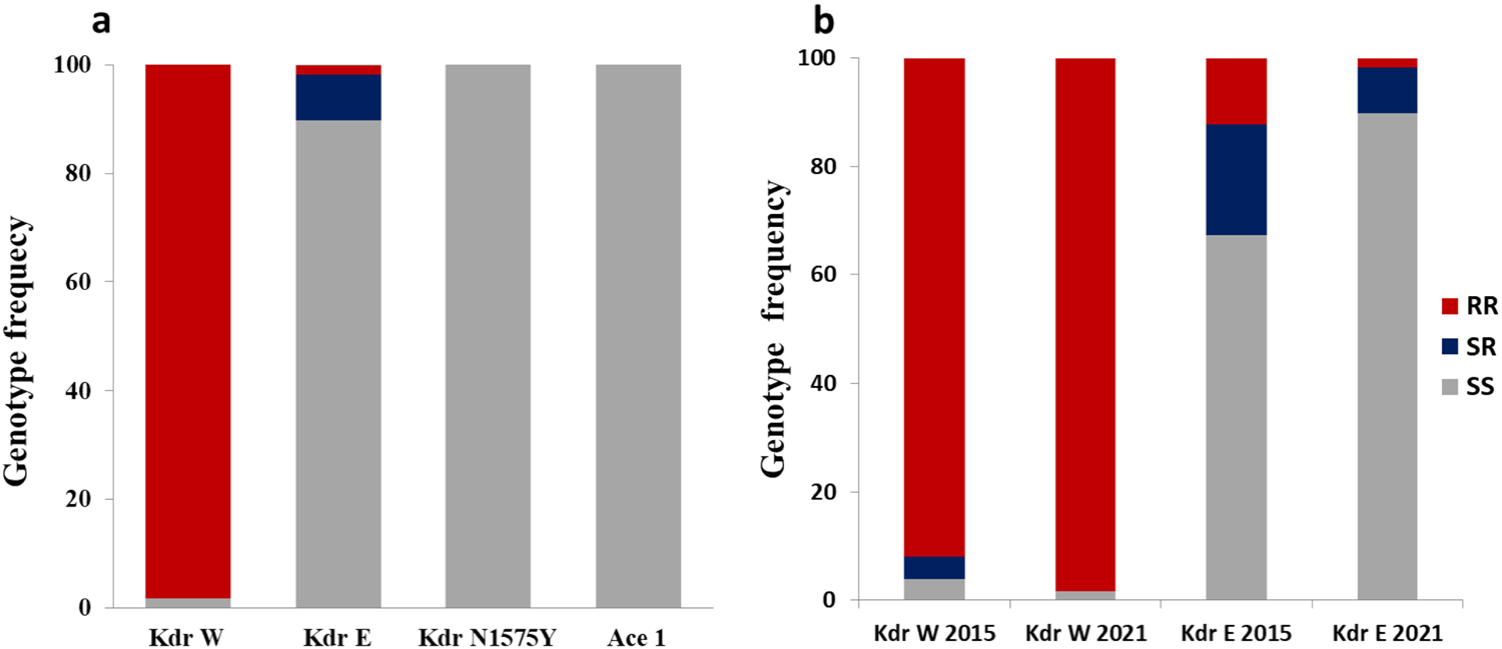
Genotype distribution for key resistance markers (**a**) and genotype comparison between *Anopheles gambiae* F0 female collected 2015 and 2021 (**b**).

The L1014F-*kdrw* mutation was close to fixation in NDjili population with 98.3% of homozygous resistant individuals (RR) and 1.7% homozygous susceptible ones (SS). No difference was obtained with samples collected in 2015 (94% RR and 6% SS). However, the L1014S-*kdre* resistant (R) allele was low (6%) with 89.9% SS, 8.4% RS and 1.7% RR (Fig. 5a). The *kdr* East susceptible allele frequency has significantly increased in 2021 (94% (2021) vs. 77.5% (2015); *P* = 0.004) (Fig. 5b). The N1575Y-*kdr* mutation associated with pyrethroid resistance and the G119S-Ace1 mutation conferring carbamate and organophosphate resistance were completely absent (Fig. 5a).

#### Transcriptional profiling of metabolic resistance genes in *A. funestus* s.s.

**Figure 6:**
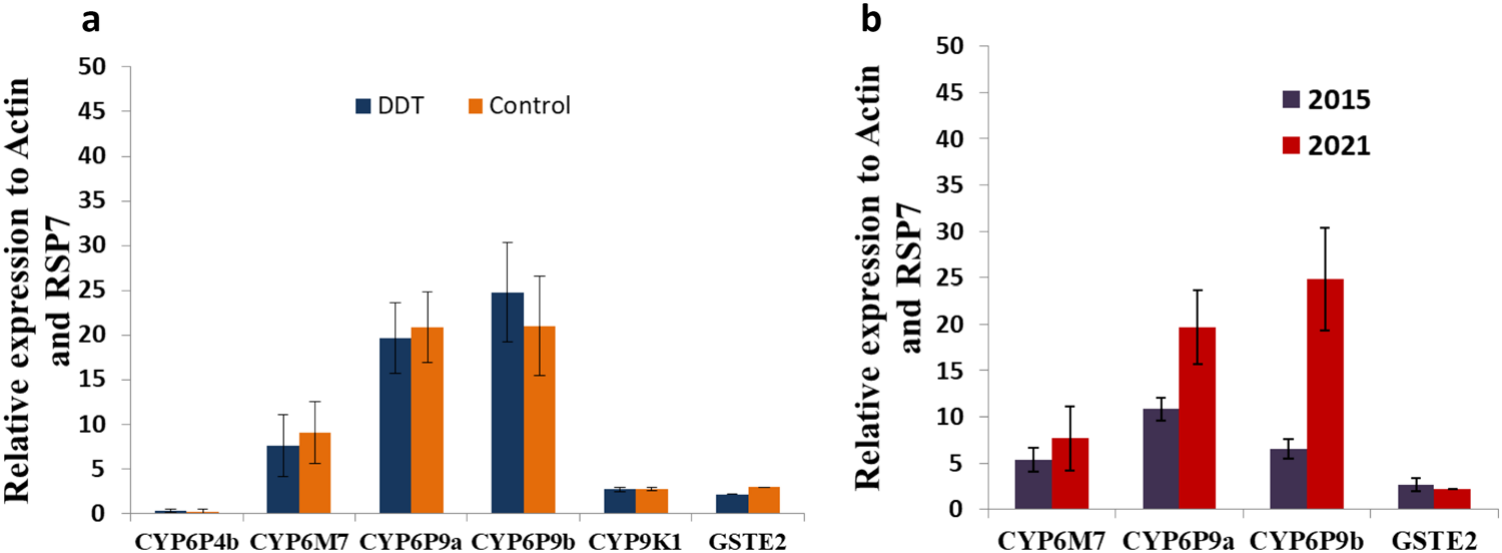
Differential gene expression of the P450 genes CYP6P9a, CYP6P9b, CYP9K1, CYP6M7 and CYP6P5 and the Gluthatione S-tranferase GSTe2 in *Anopheles funestus* from Ndjili brasserie (**a**) and Comparison of gene expression between mosquitoes collected in 2015 and 2021 (**b**). Error bars represent standard error of the mean.

The cytochrome P450 genes *CYP6P9a, CYP6P9b, CYP6M7*, *CYP9K1* and the glutathione s-transferase *GSTe2* previously shown to be conferring pyrethroid resistance in *A. funestus*^12–14^ were significantly over-expressed in DDT and permethrin resistant mosquitoes of Kinshasa compared to the susceptible FANG strain. A respective fold change of 19.69, 24.81, 7.69, 2.72, and 2.202 was obtained for these genes whereas the other P450, *CYP6P4b*, was rather downregulated with a fold change of 0.3 (Fig. 6a). Furthermore, expression level of *CYP6M7*, *GSTe2* and P450 duplicated genes *CYP6P9a* and *CYP6P9b* was significantly higher in *A. funestus* collected 2021 compared to in 2015 especially for *CYP6P9a* and *CYP6P9b* (*P* < 0.001) (Fig. 6b).

**Figure 7:**
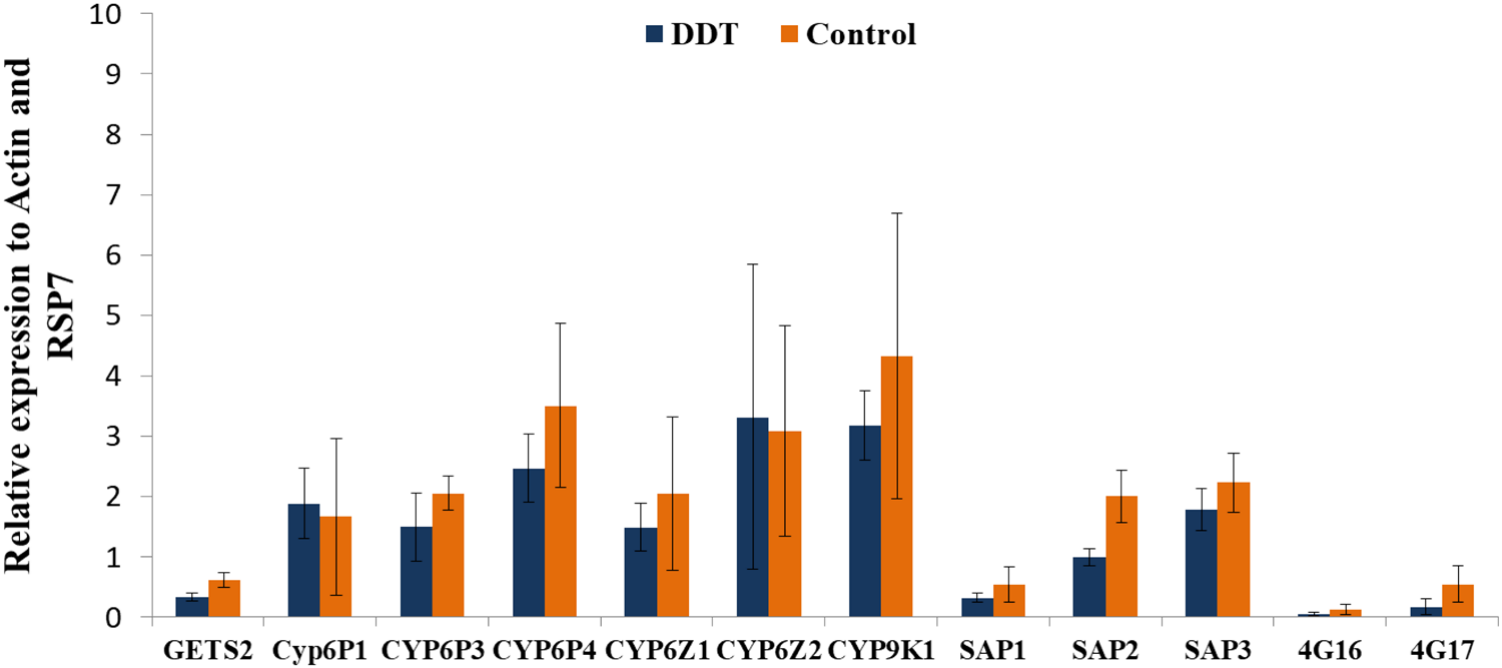
Differential gene expression in *Anopheles gambiae* from Ndjili brasserie. Error bars represent standard error of the mean.

For *A. gambiae*, the transcription pattern of *CYP4G16* and *CYP4G17*, (associated with cuticular resistance) *SAP1, SAP2, SAP3, GSTe2, CYP6P1, CYP6P3, CYP6P4, CYP6Z1, CYP6Z2, CYP9K1* and *CYP6M2,* evolving in P450 resistance were assessed and most of them were down- regulated (Fig. 7).

## Discussion

The increase intensity in insecticide resistance in *Anopheles* vectors across Africa is threatening the effectiveness of the vector control tools. The extent of this resistance and the major molecular drivers were investigated in the Capital city of DRC revealing key findings in both major vectors *A. funestus* and *A. gambiae*.

### *Plasmodium* infection rate

The two major malaria vectors were predominant (*A. gambiae* s.l., and *A. funestus* ss) in this location of Kinshasa. High *Plasmodium* infection rate was observed in *A. gambiae* (30.25%) and lower in *A. funestus* (9%) suggesting that malaria transmission is actually driven mainly by *An gambiae* in this area. This result is similar to that obtained by Riveron et al.^5^ in DRC regarding the *Plasmodium* infection rate of *A. gambiae* during the rainy season but the *Plasmodium* infection rate observed in *A. funestus* was smaller than that observed in 2015 in the same location^5^. This greater infection rate in *A. gambiae* could be associated with the higher intensity of resistance in this species with for example low mortality rates observed even at 5× (46.92% ± 3.38%) and 10× (69.38 ± 3.34%) whereas higher mortalities were observed in *A. funestus*. Furthermore, with *A. gambiae*, the infection rate observed in this study was higher than those obtained in Cameroon (20%)^15^. Overall, the results reveal the high malaria transmission rate in Kinshasa, and corroborate that DRC is a highly endemic country with 12% of all malaria cases worldwide in 2021^1^.

### Increasing resistance intensity in both *A. gambiae* and A. funestus

Overall, the bioassay results showed a significantly increased in resistance to pyrethroids and DDT in *A. funestus* and *A. gambiae* in 2021 compared to 2015 profiles. This high resistance level against pyrethroids is likely driven by increasing insecticide pressures due to LLIN-based control interventions on roof of the local practice of agriculture. The same trend was observed in *A. funestus* with an increased resistance between 2015 and 2021 with the mortality reducing from 64.7 to 43.15% for permethrin and from 87.7 to 28.7% for deltamethrin. A similar increase resistance was seen against DDT with mortality reducing from 33.8 to 18.6%^5^. Interestingly no resistance was observed to organophosphates and carbamates as already reported back in 2015 suggesting that these insecticide classes could be alternatively used for IRS in this location. A similar level of increase resistance to pyrethroids and susceptibility to carbamates was also recently reported in Uganda (Tchouakui et al. 2020) and Cameroon^15^. This increase in level of insecticide resistance could be due to the massive distribution of LLINs in DRC (PermaNet 2.0, DawaPlus 2.0 in Kinshasa, 2016; Dawa + 2.0 and Yorkool in Kongo central, 2017)^9^. In addition, Ndjili-brasserie is also located in an area of intensive agriculture with massive use of pesticides, which could be another factor driving the increased level of resistance in this area.

*A. funestus* and *A. gambiae* populations were both resistant to type I and II pyrethroids at all diagnostic concentrations of 1×, 5×, and 10×. The high resistance to pyrethroids at all diagnostic doses in *A. funestus* is similar to the observations made in Uganda a neighbouring country of the DRC^10^ and Malawi^2^. On the other hand, the resistance escalation observed in *A. gambiae* is similar to results made in DRC^9^ and different from the results obtained by Tchouakui et al.^10^ in Uganda where these mosquitoes were resistant to 1× and 5× and susceptible to 10×. This study showed a major contrast in the *A. gambiae* after pre-exposure to PBO, with a full (or nearly) recovery of susceptibility observed for type II (deltamethrin and alphacypermethrin) but only a moderate recovery with Type I (permethrin). This is similar to observation made in 2015 by Riveron et al.^5^. This variation in recovery rate suggests a difference in resistance mechanisms between these pyrethroids with P450 genes likely playing a greater role in type II (deltamethrin) than type I (permethrin). This suggests that LLINs combining deltamethrin and PBO would be more effective against this *A. gambiae* resistant population.

### Drastic loss of bio‑efficacy of LLINs between 2015 and 2021

The results of the cone test showed a low efficacy of all pyrethroid-only LLINs tested against *A. gambiae*. The low efficacy of these bednets corroborates the high pyrethroid resistance results observed in this *A. gambiae* population using WHO bioassays. This increase in resistance intensity for most of the insecticide-treated bed nets (ITNs) tested is greater than those obtained across Africa by Riveron et al. in DRC^5^, Menze et al*.* in Cameroon^2^, and Tchouakui et al. in Uganda^10^. This loss of bed net efficacy may be due to selection pressure induced by massive distribution of bed nets by the government^9^ and observation of the massive use of pesticide in farming in this area. Even the net with synergist PBO combined with permethrin (Olyset Plus), induced a low mortality with *A. gambiae*, indicating that P450 genes may not be the main drivers of the permethrin resistance observed in DRC, but rather other mechanisms such the *kdr* mutation, which is nearly fixed in this population or cuticular resistance^5^.

The same pattern of low efficacy of bednet was observed in *A. funestus* except for Olyset Plus (containing PBO) which showed higher efficacy with 100% mortality.

### Increasing allele and genotype frequencies between 2015 and 2021 contribute to increasing pyrethroid resistance aggravation

The L1014F (*kdrw*) mutation conferring insecticide resistance to permethrin and DDT in *A. gambiae* was closed to fixation in Kinshasa mosquito population. This result corroborates the extremely high resistance observed with permethrin and DDT in *A. gambiae* population. The same mutation has been previously detected in Kinshasa with the high frequency of the L1014F *kdrw* mutation (87.8%) by^5^. In addition, this mutation has been detected in other location in DRC^4,16^.

The 1014S resistance allele was recorded at the frequency of 10.1% in mosquito population that contributes to maintain the high resistance level to pyrethroids and DDT. Furthermore, the N1575Y mutation was still not detected in the Kinshasa samples as previously by Jones et al.^17^.

The G119S-Ace-1 mutation conferring bendiocarb resistance in *A. gambiae* s.l. was also not found in Kinshasa as it was already the case in 2015^5^. This absence of the 119S Ace-1^R^ supports the susceptibility to carbamates and organophosphates seen in this population. However, because this mutation was recently detected at a low frequency (0.11–0.19) in *A. gambiae* in eastern DRC^18^ it is important to continue the monitoring. However, Metabolic mechanisms are more likely involved and a cost linked to this mutation. In *A. funestus* s.s the high resistance to DDT corroborates the high frequency of L119F-GSTe2 resistant allele in Ndjili mosquito population, confirming the results obtained by Riveron et al.^5^ in this same mosquito population. This would further support the implication that the L119F-GSTe2 marker in the metabolic resistance to DDT/permethrin. On the other hand, the A296S-RDL GABA receptor mutation known to confer resistance to dieldrin^19^, was observed with high allelic frequency of 56.25% but lower than the 66.7% observed Riveron et al.^5^ which confirm the past or reduced use of this insecticide in agricultural area. Such reduction could be explained by a fitness cost associated to this allele as commonly seen for resistance alleles^10,20^. On the other hand, the extremely low frequency of resistant CYP6P9a, CYP6P9b and CYP6P9a/b alleles observed in this study contrasts with the high frequency of this allele observed in the eastern DRC mosquito population^11,18^. This implies that migration and gene flow could be responsible for the presence of this mutation in the Kinshasa population, as the resistant allele is found in the south and part of East Africa and completely absent from other parts of Africa^21^. However, the first detection of CYP6P9a/b_R alleles in Kinshasa even though at low frequency, suggest that these alleles have now migrated to West DRC and could further exacerbate resistance in the coming years as seen in southern Africa^13^.

### Transcription profile of resistance genes in *A. funestus*

The transcription analyses show that, the *CYP6P9a*, *CYP6P9b*, and *CYP6M7* genes, known to be involved in pyrethroid resistance in *A. funestus* genes^12,13,22^ are significantly up-regulated in our field *A. funestus* s.s mosquito population (alive and unexposed to permethrin) compared to susceptible strain Fang. This expression result is higher than the one obtained by^5^ and could explain the resistance escalation to pyrethroids observed in this location. Twelve (12) candidates genes were analysed in *A. gambiae* including, *Cyp6Z2, Cyp6P4*, *CYP9K1* which had more than twofold-changes compared to the susceptible Kisumu strain; which is not surprizing and not different from the microarray results obtained by Nardini et al. in DRC^3^ regarding *CYP9K1* gene.

## Methods

### Study area and mosquito collection

Adult *Anopheles* female mosquitoes were collected inside households using electric aspirators at Ndjili- Brasserie, a suburb of Kinshasa (4° 19′ 39″ S, 15° 18′ 48″ E), in July 2021 in the same houses as done in May 2015^5^. *Anopheles* females mosquitoes collected were morphologically identified as belonging to *A. funestus* group or *A. gambiae* s.l complex according to morphological keys^23^. These mosquitoes were kept 4–5 days in paper cups and fed with sugar until they became fully gravid and forced to lay eggs individually in 1.5 mL Eppendorf tubes. After egg hatching, larvae were placed in trays and reared to adults mosquitoes as previously described^24^.

### Identification of mosquito species

Whole mosquitoes (*A. funestus* s.l. and *A. gambiae* s.l.) collected in Ndjili-Brasserie, were used for genomic DNA extraction using the Livak protocol^25^. Members of the *A. funestus* s.l. group were identified using the cocktail polymerase chain reaction (PCR) assay^26^, whereas the SINE PCR protocol was used to identify those from the *A. gambiae, A. coluzzii and A. arabiensis*^27^.

### Plasmodium infection rate determination

The *Plasmodium* infection rate was estimated using gDNA of the whole body of mosquito to detect the presence of *P. falciparum* (F+) and/or *P. ovale*, *P. vivax* and *Plasmodium malariae* (OVM+) in 60 *A. gambiae* s.l. and 60 *A. funestus* sensu stricto (s.s.) field-collected F_0_ females individually using the TaqMan assay, as previously described^28,29^. We used nested PCR assay^30^ to confirm the results of TaqMan assay and differentiate between the species belonging to the OVM+.

### Insecticide susceptibility assays

The susceptibility patterns of both *A. funestus* s.s. and *A. gambiae* s.l. to various insecticides were performed using the F_1_ generation following the WHO protocol^31^. Insecticides tested include pyrethroids: permethrin (0.75%), deltamethrin (0.05%) and α-cypermethrin (0.05%), carbamate: bendiocarb (0.1%), organochlorine: dichlorodiphenyltrichloroethane (DDT) (4%) and the organophosphate: pirimiphos-methyl (0.25%) (papers were obtained from the Universiti Sains Malaysia).

All the tests were performed at standard insectary conditions of 25 ± 2 °C temperature and 70–80% relative humidity. For each test, at least three replicates of 20–25 F_1_ female mosquitoes of 2–5 day-old were exposed to insecticide-impregnated papers for 1 h and control mosquitoes were exposed to non-impregnated papers. After the exposure, mosquitoes were transferred to a holding tube provided with cotton soaked in a 10% sugar solution. The knockdown was recorded 60 min after exposure to insecticide and mortality was determined 24 h later.

Based on the results of resistance status with 1× (discriminating concentration (DC)) of pyrethroid (permethrin and deltamethrin), intensity bioassays were carried out with 5× DC and 10× DC of these insecticides. The intensity bioassays with 5× and 10× DC were performed following the WHO 2016 test procedure^31^.

### PBO synergist assays

In order to investigate the potential role of cytochrome P450s genes in the observed resistance, *A. gambiae* s.l. females were pre-exposed to 4% piperonyl butoxide (PBO) for 60 min and immediately exposed to permethrin (0.75%), deltamethrin (0.05%) and α-cypermethrin (0.05%). The mortality was recorded after 24 h and compared with the mortality obtained for mosquitoes not pre-exposed to PBO using unpaired Student *t* test.

### Insecticide-treated bed nets efficacy assays

The efficacy of the LLINs was estimated by 3-min exposure cone bioassays following the WHO guidelines^32^. The nets tested for *A. gambiae* included Olyset, Olyset Plus, PermaNet 2.0, PermaNet 3.0-side and –roof, Royal guard, and an untreated net (as a control). Due to the low number of *A. funestus*, only Olyset, Olyset Plus, PermaNet 2.0 and PermaNet 3.0–roof were tested. As done in 2015, five replicates of 10 *A. gambiae* F_1_ female (2–5 days old) were placed in plastic cones enclosed with the mosquito net for 3 min. But for *A. funestus* due to the very low number of mosquitoes, only three replicates with 5 mosquitoes/cone were tested. Mosquitoes were then transferred in small holding paper cups with cotton soaked in a 10% sugar solution. Mortality was determined 24 h after exposure. The assay was carried out at temperature of 25 °C ± 2 °C and 80% ± 10% relative humidity.

### Genotyping of resistance markers in *A. funestus* s.s.

Changes in allele frequency between 2015 and 2021 was assessed in *A. funestus* s.s including the L119F-GSTe2 (DDT/permethrin), A296S-RDL (dieldrin), *CYP6P9a*, *CYP6P9b* and 6.5 kb-SV (pyrethroids). The A296S-RDL mutation was genotyped using TaqMan assays and allele-specific PCR (AS-PCR) was used to genotype the L119F-GSTe2 as previously described^33^. Whereas the presence of the CYP6P9a/b_R allele was assessed using PCR–RFLP, while the 6.5 kb-SV was genotyped using a multiplex PCR Assay recently designed^11,21,34^.

### Genotyping of resistance markers in *A. gambiae* s.l

The L1014F-*drw* mutation, the L1014S-*kdre* and the N1575Y responsible for DDT and pyrethroid resistance in *A. gambiae* s.l. and the G119S *ace*-*1* conferring organophosphate and carbamate resistance in this species was genotyped using TaqMan assays with two labelled fluorochromes probes FAM and HEX^28^.

### Transcription profile of resistance genes in *A. funestus* s.s and *A. gambiae* s.l.

Total RNA was extracted from 3 batches of 10 F_1_ female *A. funestus* s.s. and *A. gambiae* s.l. mosquitoes non exposed to insecticides and the FANG and KISUMU susceptible strain, as previously described^17,35^.

In *A. funestus* s.s the transcription patterns of *CYP9K1*, *CYP6P4b*, *CYP6P9a*, *CYP6P9b*, *GSTe2* and *CYP6M7*, major pyrethroid resistance genes^14^ were assessed by a quantitative reverse transcription PCR (qRT-PCR).

In *A. gambiae*, the transcription patterns of *SAP1*, *SAP2*, *SAP3*, *CYP4G16*, *CYP4G17* (cuticular resistance), *GSTe2*, *CYP6P1*, *CYP6P3*, *CYP6P4*, *CYP6Z1*, *CYP6Z2*, *CYP9K1* and *CYP9M2*, involving in metabolic resistance were also assessed. After normalization with housekeeping genes *Actin* (AFUN006819) and *RSP7* (AFUN007153-RA) for *A. funestus*, *Elongation Factor* (AGAP009441) and *RSP7* for *A. gambiae*, the relative expression for each gene was calculated according to the 2^−ΔΔCT^ method^36^. The statistical significance between gene expression estimates was performed using unpaired Student *t* test. The expression level of these genes in 2021 was compared with that of 2015.

## Conclusions

By revisiting the resistance patterns of the same population of malaria vectors after six years, this study has exhibited multiple evidences of an intensification of pyrethroid resistance in both *A. gambiae* and *A. funestus* in the capital of the Democratic Republic of Congo. This resistance aggravation is greater in *A. gambiae* while also signifiant in *A. funestus* and impacts the efficacy of LLINs notably pyrethroid-only nets. The resistance escalation is supported by increased frequency of major resistance alleles or first detection of previously absent ones. Altogether this study makes a strong case for deploying novel control interventions that rely on new generations LLINs such as PBO-based nets or dual-active nets such as Interceptor G2 as well as consideration of IRS using organophosphates or new insecticides (neonicotinoids) to boost vector control efforts in this high malaria burden country.

## Data Availability

All the data are present in the manuscript.

## References

[1] WHO. *World Malaria Report 2021* (2021).

[2] Menze BD (2022). Marked aggravation of pyrethroid resistance in major malaria vectors in Malawi between 2014 and 2021 is partly linked with increased expression of P450 alleles. BMC Infect. Dis..

[3] Nardini L (2017). Malaria vectors in the Democratic Republic of the Congo: The mechanisms that confer insecticide resistance in *Anopheles gambiae* and *Anopheles funestus*. Malar. J..

[4] Basilua Kanza JP (2013). Pyrethroid, DDT and malathion resistance in the malaria vector *Anopheles gambiae* from the Democratic Republic of Congo. Trans. R. Soc. Trop. Med. Hyg..

[5] Riveron JM (2018). High plasmodium infection rate and reduced bed net efficacy in multiple insecticide-resistant malaria vectors in Kinshasa, Democratic Republic of Congo. J. Infect. Dis..

[6] Martinez-Torres D (1998). Molecular characterization of pyrethroid knockdown resistance (kdr) in the major malaria vector *Anopheles gambiae* ss. Insect Mol. Biol..

[7] Ranson H (2000). Identification of a point mutation in the voltage-gated sodium channel gene of Kenyan *Anopheles gambiae* associated with resistance to DDT and pyrethroids. Insect Mol. Biol..

[8] Ranson H (2011). Pyrethroid resistance in African anopheline mosquitoes: What are the implications for malaria control?. Trends Parasitol..

[9] Wat’senga F (2020). Intensity of pyrethroid resistance in *Anopheles gambiae* before and after a mass distribution of insecticide-treated nets in Kinshasa and in 11 provinces of the Democratic Republic of Congo. Malar. J..

[10] Tchouakui M (2021). Pyrethroid resistance aggravation in Ugandan malaria vectors is reducing bednet efficacy. Pathogens.

[11] Mugenzi LM (2020). A 6.5-kb intergenic structural variation enhances P450-mediated resistance to pyrethroids in malaria vectors lowering bed net efficacy. Mol. Ecol..

[12] Ibrahim SS (2015). Allelic variation of cytochrome P450s drives resistance to bednet insecticides in a major malaria vector. PLoS Genet..

[13] Riveron JM (2014). The highly polymorphic CYP6M7 cytochrome P450 gene partners with the directionally selected CYP6P9a and CYP6P9b genes to expand the pyrethroid resistance front in the malaria vector *Anopheles funestus* in Africa. BMC Genom..

[14] Sandeu MM (2020). A differential expression of pyrethroid resistance genes in the malaria vector *Anopheles funestus* across Uganda is associated with patterns of gene flow. PLoS ONE.

[15] Menze BD (2018). Bionomics and insecticides resistance profiling of malaria vectors at a selected site for experimental hut trials in central Cameroon. Malar. J..

[16] Bobanga T (2013). Field efficacy and acceptability of PermaNet® 3.0 and OlysetNet® in Kinshasa, Democratic Republic of the Congo. J. Vector Borne Dis..

[17] Jones CM (2012). Footprints of positive selection associated with a mutation (N1575Y) in the voltage-gated sodium channel of *Anopheles gambiae*. Proc. Natl. Acad. Sci..

[18] Bandibabone J (2021). Investigating molecular mechanisms of insecticide resistance in the Eastern Democratic Republic of the Congo. Malar. J..

[19] Wondji CS (2011). Identification and distribution of a GABA receptor mutation conferring dieldrin resistance in the malaria vector *Anopheles funestus* in Africa. Insect Biochem. Mol. Biol..

[20] Tchouakui M (2022). Comparative study of the effect of solvents on the efficacy of neonicotinoid insecticides against malaria vector populations across Africa. Infect. Dis. Poverty.

[21] Weedall GD (2019). A cytochrome P450 allele confers pyrethroid resistance on a major African malaria vector, reducing insecticide-treated bednet efficacy. Sci. Transl. Med..

[22] Riveron JM (2013). Directionally selected cytochrome P450 alleles are driving the spread of pyrethroid resistance in the major malaria vector *Anopheles funestus*. Proc. Natl. Acad. Sci..

[23] Gillies M, Coetzee M (1987). A supplement to the Anophelinae of Africa South of the Sahara. Publ. S. Afr. Inst. Med. Res..

[24] Morgan JC (2010). Pyrethroid resistance in an *Anopheles funestus* population from Uganda. PLoS ONE.

[25] Livak KJ (1984). Organization and mapping of a sequence on the Drosophila melanogaster X and Y chromosomes that is transcribed during spermatogenesis. Genetics.

[26] Koekemoer L (2002). A cocktail polymerase chain reaction assay to identify members of the *Anopheles funestus* (Diptera: Culicidae) group. Am. J. Trop. Med. Hyg..

[27] Santolamazza F (2008). Insertion polymorphisms of SINE200 retrotransposons within speciation islands of *Anopheles gambiae* molecular forms. Malar. J..

[28] Bass C (2008). PCR-based detection of Plasmodium in Anopheles mosquitoes: A comparison of a new high-throughput assay with existing methods. Malar. J..

[29] Mulamba C (2014). Contrasting Plasmodium infection rates and insecticide susceptibility profiles between the sympatric sibling species *Anopheles parensis* and *Anopheles funestus* ss: A potential challenge for malaria vector control in Uganda. Parasit. Vectors.

[30] Snounou G (1993). Identification of the four human malaria parasite species in field samples by the polymerase chain reaction and detection of a high prevalence of mixed infections. Mol. Biochem. Parasitol..

[31] WHO (2016). World Malaria Report 2015.

[32] World Health Organization (2013). Guidelines for Laboratory and Field-Testing of Long-Lasting Insecticidal Nets.

[33] Tchouakui M (2019). A marker of glutathione S-transferase-mediated resistance to insecticides is associated with higher Plasmodium infection in the African malaria vector *Anopheles funestus*. Sci. Rep..

[34] Mugenzi LM (2019). Cis-regulatory CYP6P9b P450 variants associated with loss of insecticide-treated bed net efficacy against *Anopheles funestus*. Nat. Commun..

[35] Riveron JM (2015). Rise of multiple insecticide resistance in *Anopheles funestus* in Malawi: A major concern for malaria vector control. Malar. J..

[36] Schmittgen TD, Livak KJ (2008). Analyzing real-time PCR data by the comparative CT method. Nat. Protoc..

